# Supplementary material for: Co-Infection of Culex tarsalis Mosquitoes with Rift Valley Fever Phlebovirus Strains Results in Efficient Viral Reassortment
Source: Viruses. 2025 Jan 11;17(1):88. doi: 10.3390/v17010088 (PMC11768849; doi:10.3390/v17010088)
Supplement: Supplementary file 1 [file viruses-17-00088-s001.zip › viruses-3359389-supplementary.pdf]

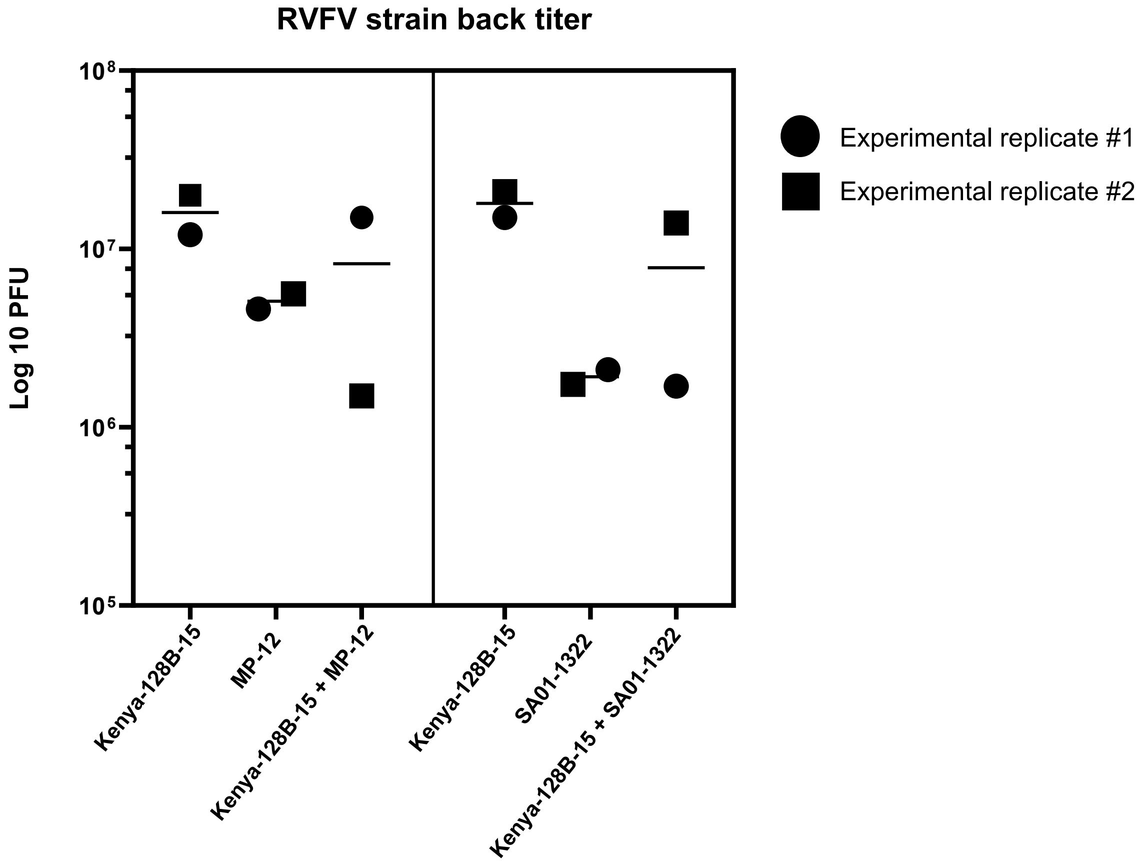

Supplementary Figure S1. Rift Valley fever virus strain back titration of individual input virus harvested from Vero cells and dual-infected bloodmeals use to generate infected *Culex tarsalis*. Data demonstrate viral titers for experimental replicate 1 (circles) and 2 (squares). Viral infected cells were generated by infecting cells with MOI 0.01 for each strain. At 72 hours post-infection (hpi) virus was harvested and mixed at a 1:1:1 ratio for each viral strain to bovine blood. A representative aliquot of generated bloodmeal was incubated at 37°C for approximately the same time mosquitoes were permitted to feed. Horizontal line indicates mean.

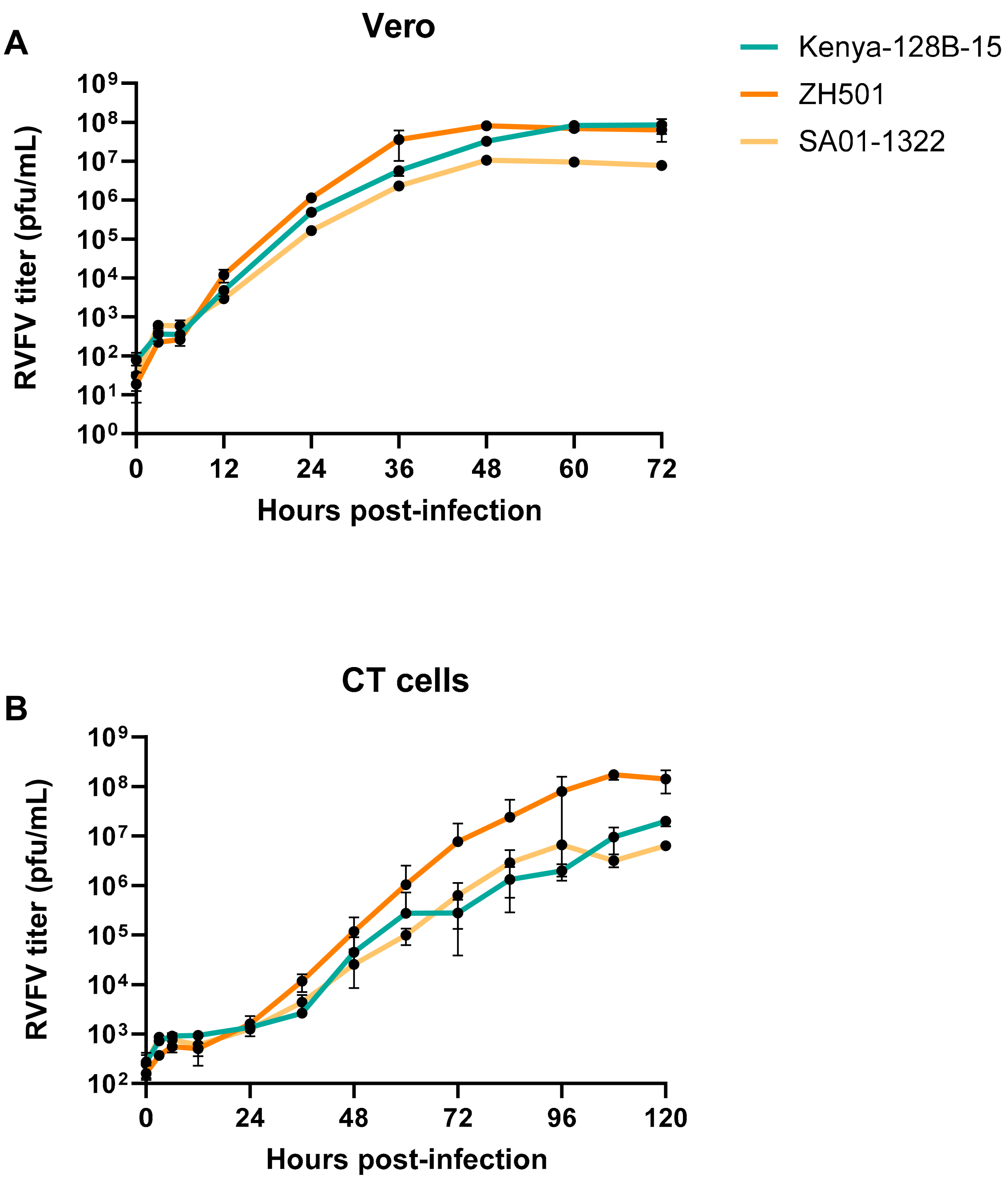

Supplementary Figure S2. Growth kinetics of RVFV isolates in mammalian and mosquito backgrounds. RVFV isolates Kenya-128B-15, ZH501, and SA01-1322 were used to infect either Vero (A) or *Cx. tarsalis*-dervied (CT) cells. Supernatant containing virus was collected from infected flasks at various time points post-infection and plaque forming units determined. Concentration of virus represented as the Log10 titer of plaque forming units per mL. Black dots indicate mean viral titer for each time point across three replicates. Error bars represent the standard error of the mean.
